# Alzheimer’s disease-associated (hydroxy)methylomic changes in the brain and blood

**DOI:** 10.1186/s13148-019-0755-5

**Published:** 2019-11-27

**Authors:** Roy Lardenoije, Janou A. Y. Roubroeks, Ehsan Pishva, Markus Leber, Holger Wagner, Artemis Iatrou, Adam R. Smith, Rebecca G. Smith, Lars M. T. Eijssen, Luca Kleineidam, Amit Kawalia, Per Hoffmann, Tobias Luck, Steffi Riedel-Heller, Frank Jessen, Wolfgang Maier, Michael Wagner, René Hurlemann, Gunter Kenis, Muhammad Ali, Antonio del Sol, Diego Mastroeni, Elaine Delvaux, Paul D. Coleman, Jonathan Mill, Bart P. F. Rutten, Katie Lunnon, Alfredo Ramirez, Daniël L. A. van den Hove

**Affiliations:** 1 School for Mental Health and Neuroscience (MHeNS), Department of Psychiatry and Neuropsychology, Maastricht University, P.O. Box 616, 6200 MD Maastricht, the Netherlands; 2 Department of Psychiatry and Psychotherapy, University Medical Center Göttingen, 37075 Göttingen, Germany; 3 University of Exeter Medical School, University of Exeter, Exeter, UK; 4 Division of Neurogenetics and Molecular Psychiatry, Department of Psychiatry and Psychotherapy, University of Cologne, Medical Faculty, 50937 Cologne, Germany; 5 Department of Neurodegeneration and Gerontopsychiatry, University of Bonn, 53127 Bonn, Germany; 6 Department of Bioinformatics—BiGCaT, Maastricht University, Maastricht, The Netherlands; 7 German Center for Neurodegenerative Diseases (DZNE), 53127 Bonn, Germany; 8 Institute of Human Genetics, University of Bonn, 53127 Bonn, Germany; 9 Department of Genomics, Life & Brain Center, University of Bonn, 53127 Bonn, Germany; 10 Division of Medical Genetics, University Hospital and Department of Biomedicine, University of Basel, CH-4058 Basel, Switzerland; 11 Institute of Social Medicine, Occupational Health and Public Health, University of Leipzig, 04103 Leipzig, Germany; 12 Department of Psychiatry and Psychotherapy, University of Cologne, Medical Faculty, 50937 Cologne, Germany; 13 Department of Psychiatry and Division of Medical Psychology, University of Bonn, 53105 Bonn, Germany; 14 Luxembourg Centre for Systems Biomedicine (LCSB), University of Luxembourg, Esch-sur-Alzette, Luxembourg; 15 Moscow Institute of Physics and Technology, Dolgoprudny, Moscow, Russian Federation; 16 CIC bioGUNE, Bizkaia Technology Park, 801 Building, 48160 Derio, Spain; 17 IKERBASQUE, Basque Foundation for Science, Dolgoprudny Bilbao, Spain; 18 L.J. Roberts Center for Alzheimer’s Research Banner Sun Health Research Institute, Sun City, AZ USA; 19 Biodesign Institute, Neurodegenerative Disease Research Center, Arizona State University, Tempe, AZ USA; 20 Institute of Psychiatry, King’s College London, London, UK; 21 Department of Psychiatry, Psychosomatics and Psychotherapy, University of Würzburg, Würzburg, Germany

**Keywords:** Alzheimer’s disease, Epigenetics, DNA methylation, DNA hydroxymethylation, Brain, Middle temporal gyrus, Blood

## Abstract

**Background:**

Late-onset Alzheimer’s disease (AD) is a complex multifactorial affliction, the pathogenesis of which is thought to involve gene-environment interactions that might be captured in the epigenome. The present study investigated epigenome-wide patterns of DNA methylation (5-methylcytosine, 5mC) and hydroxymethylation (5-hydroxymethylcytosine, 5hmC), as well as the abundance of unmodified cytosine (UC), in relation to AD.

**Results:**

We identified epigenetic differences in AD patients (*n* = 45) as compared to age-matched controls (*n* = 35) in the middle temporal gyrus, pertaining to genomic regions close to or overlapping with genes such as *OXT* (− 3.76% 5mC, *p*_*Šidák*_ = 1.07E−06), *CHRNB1* (+ 1.46% 5hmC, *p*_*Šidák*_ = 4.01E−04), *RHBDF2* (− 3.45% UC, *p*_*Šidák*_ = 4.85E−06), and *C3* (− 1.20% UC, *p*_*Šidák*_ = 1.57E−03). In parallel, in an independent cohort, we compared the blood methylome of converters to AD dementia (*n* = 54) and non-converters (*n* = 42), at a preclinical stage. DNA methylation in the same region of the *OXT* promoter as found in the brain was found to be associated with subsequent conversion to AD dementia in the blood of elderly, non-demented individuals (+ 3.43% 5mC, *p*_*Šidák*_ = 7.14E−04).

**Conclusions:**

The implication of genome-wide significant differential methylation of *OXT*, encoding oxytocin, in two independent cohorts indicates it is a promising target for future studies on early biomarkers and novel therapeutic strategies in AD.

## Background

The neuropathological cascade of the world’s leading cause of dementia, late-onset Alzheimer’s disease (AD), is characterized by the progressive accumulation of extracellular amyloid plaques and intracellular neurofibrillary tangles, followed by neuronal cell death. The susceptibility to AD is determined by the complex interaction of genetic, environmental, and life-style factors, as well as epigenetic factors. Genetic research has been successful in identifying genetic variants modulating susceptibility to AD, including the first and strongest genetic risk factor for AD in the *APOE* gene. In addition to *APOE*, large-scale genome-wide association studies looking at AD have identified a number of independent common variants with a small-to-modest effect size [1]. Besides genetics, recent studies have suggested an important role for epigenetic mechanisms in the etiology of AD [2], with reports of both global and gene-specific alterations in epigenetic modifications [3–6].

**Fig. 1:**
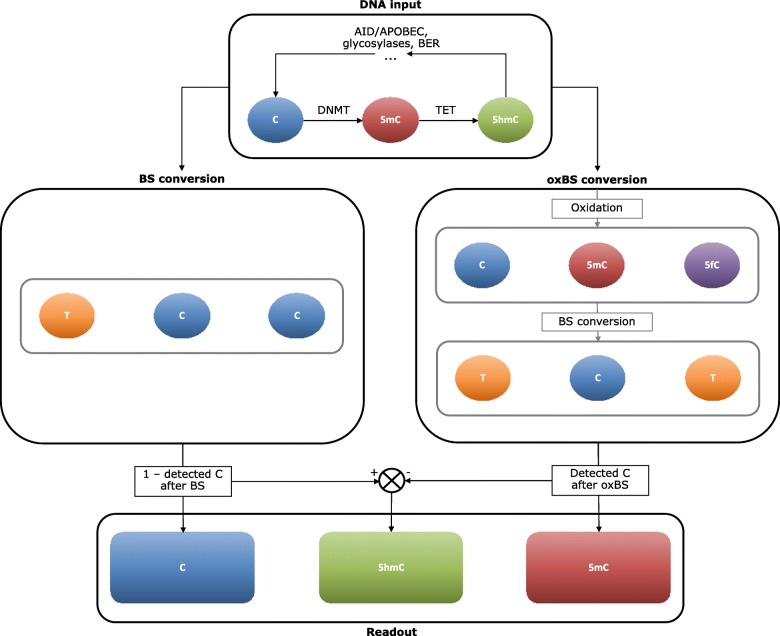
Overview of the procedure to detect unmodified cytosines (C), 5-methylcytosine (5mC), and 5-hydroxymethylcytosine (5hmC). Naturally, C can be converted to 5mC by DNA methyltransferases (DNMTs) and 5mC can be oxidized by ten-eleven translocation (TET) enzymes, resulting in 5hmC. There are several proposed demethylation pathways through which 5mC and 5hmC can be converted back to C. DNA samples were split in two, one half was only treated with bisulfite (BS), which converts C into thymine (T). 5mC and 5hmC are protected against this conversion, and will be read as a C on the array. The detected C signal after BS conversion is thus actually the combined 5mC and 5hmC signal. As the signals are converted to fractions, with C + 5mC + 5hmC = 1, the fraction of C in the input DNA can be determined by subtracting the C signal after BS conversion (representing the combined 5mC and 5hmC fraction in the input DNA) from 1. The other half of the DNA sample was first oxidized, which converts 5hmC into 5-formylcytosine (5fC), and then treated with BS. 5fC is not protected against the BS conversion, so it also turns into T. C detected on the array after this oxidative BS (oxBS) conversion thus represents the fraction of 5mC in the input DNA. The 5hmC fraction in the input DNA can be determined by subtracting the fraction of 5mC (detect C after oxBS) from the combined 5mC and 5hmC fraction (detected C after BS). This procedure results in three readout signals: unmodified C, 5mC, and 5hmC. Note that 5fC, and probably also 5-carboxylcytosine, are included in the unmodified C fraction.

Several types of epigenetic DNA modifications have been described, including DNA methylation (5-methylcytosine, 5mC) and DNA hydroxymethylation (5-hydroxymethylcytosine, 5hmC). While the best studied epigenetic DNA modification, 5mC, plays an important gene regulatory role in most tissues, 5hmC seems to have a different impact on gene expression and is particularly enriched in the brain [7, 8], where it may play an important role in learning and memory [9, 10]. Unfortunately, conventional bisulfite (BS) conversion, a widely used procedure when quantifying DNA methylation, does not distinguish between 5mC and 5hmC. However, combining measurements from BS- and oxidative BS (oxBS)-converted DNA now allows for the quantification of both 5mC and 5hmC levels (Fig. 1).

Where genetic factors can identify persons at risk for developing AD from birth, epigenetic markers may offer more dynamic views on trajectories of biological change and may therefore be able to offer an improved, chronological insight into the sequence of events at different stages of AD. As brain tissue cannot be readily sampled in living humans, blood may offer an alternative. Available research on the blood DNA methylome in relation to AD is limited and mainly focuses on the direct comparison of AD cases and healthy controls [3, 11, 12]. Identifying disease-predicting biological profiles at pre-dementia stages of AD may provide improved precision in predicting onset of dementia and give potential treatments a better timeframe to successfully impede, or even halt disease progression [13, 14].

In the present study, we explored the association between AD and epigenetic dysregulation by quantifying 5mC and 5hmC, as well as unmodified cytosine (UC) proportions [15], at a single-site resolution in middle temporal gyrus (MTG) tissue obtained from AD patients (*n* = 45) and elderly, non-demented controls (*n* = 35; see Table 1 and the “Materials and methods” section for detailed demographics) [16]. This brain region was selected as the MTG is known as a site of early AD pathology [17], and differences in global levels of DNA methylation and hydroxymethylation have previously been reported in this brain region in AD [18]. While informative on its own, the inclusion of UC measurements also allows us to better compare our findings with previous studies using conventional BS conversion, since UC is determined by subtracting the BS signal (5mC + 5hmC) from 1 (Fig. 1). Even though the effects will be opposite from directly using the BS signal, incorporating UC in our study represents a crucial legacy analysis that enables the comparison with previous studies solely relying on the BS signal. Moreover, mechanistically, as an example, the affinity of a transcription factor may be different in the presence of UC, 5mC, or 5hmC, implicating that differential levels of UC (in the absence of significantly different 5mC or 5hmC levels) may have direct functional implications on gene expression. We followed up the brain analysis exploring DNA methylation in whole blood in an independent cohort, including samples from AD-converters and non-converters at two time points, before (54 converters, 42 controls) and after (41 converters, 42 controls) conversion to clinical AD (see Table 2 and the “Materials and methods” section for detailed demographics). Blood DNA methylomic markers were measured using only BS-converted DNA, as 5hmC has a very low prevalence in blood [8].

**Table 1:** Cohort demographics—brain tissue

	AD patients	Non-demented controls
*N*	45	35
Gender(m/f)	22/23	17/18
Age of death (mean ± SD)	85.09 (6.24)	84.46 (5.50)
PMI (Mean ± SD)	2.77 (0.69)	2.87 (1.03)
Plaque total (mean ± SD)	12.97 (2.25)	4.65 (4.30)
Tangle total (mean ± SD)	11.02 (4.16)	3.96 (2.10)
Braak stage (range (median))	II–VI (V)	I–IV (III)

The brain tissue cohort consisted of 80 middle temporal gyrus (MTG) tissue samples obtained from the Banner Sun Health Research Institute (Sun City, AZ, USA), from which HM 450K array BS and oxBS data was generated. Displayed is the number of samples in each group and the distributions of gender, age, postmortem interval (hours), Braak stage, and plaque and tangle total (the sum of average Aβ plaque densities and tangle densities (resp.) in the entorhinal cortex, hippocampus, parietal lobe cortex, temporal lobe cortex and frontal lobe cortex)

**Table 2:** Cohort demographics—blood samples

	Controls	Converters
Baseline (T1)		
*N*	42	54
Gender (m/f)	10/32	17/34
Age at baseline (mean ± SD)	81.00 ± 3.11	82.31 ± 3.55
APOE4 carriers	43%	43%
Follow-up (T2)		
*N*	42	41
Gender (m/f)	10/32	13/28
Age at baseline (mean ± SD)	81.00 ± 3.11	82.01 ± 3.51
APOE4 carriers	43%	41%

Blood samples were obtained from the German Study on Ageing, Cognition and Dementia in Primary Care Patients (AgeCoDe) cohort, and HM 450K array BS data was generated. The cohort includes controls, who showed no signs at baseline or follow-up, and converters who showed no signs of dementia at baseline, but were diagnosed with AD dementia at follow-up. DNA samples were collected at baseline and follow-up for both groups. Displayed is the number of samples in each group, the distributions of Gender and Age at baseline, and the percentage of APOE ɛ4 allele carriers

## Results

### Middle temporal gyrus

Site-specific 5mC, 5hmC, and UC levels were determined for the MTG using Illumina’s Infinium HumanMethylation450K microarray (HM 450K array) with BS and oxBS-converted DNA (Fig. 1; see Tables 1 and 2 for cohort demographics). An epigenome-wide association study (EWAS) was performed for each DNA modification to identify the association with AD. The adjusted linear models showed no signs of inflation (all lambda values were between 0.95 and 1.05; see Additional file 2: Figure S1 for QQ plots). None of the AD-associated CpG sites in the MTG passed false discovery rate (FDR) correction (Additional file 1: Tables S1–S3).

A structural and functional genomic annotation enrichment analysis on the 1000 highest ranked sites indicated a significant enrichment of several CpG island features, gene features, and alternative transcription events. This included an enrichment of mainly gene body sites for the 5mC (fold enrichment = 1.42, *p* = 1.17E−10) and 5hmC (fold enrichment = 1.17, *p* = 3.64E−03) results and mainly intergenic sites for the UC (fold enrichment = 1.59, *p* = 1.67E−09) results (Additional file 2: Figure S8; Additional file 1: Table S7).

A regional analysis, looking at the spatial correlation of adjacent modified positions, detected 1 differentially methylated region (DMR), 1 differentially hydroxymethylated region (DHR), and 11 differentially unmodified regions (DURs) that were associated with AD in the MTG (Table 3; Additional file 2: Figure S3). Analysis of MTG expression data of genes annotated to DMRs, DHRs, and DURs showed a significant negative correlation between a DUR associated with *RHBDF2* and *RHBDF2* RNA expression (*ρ* = -0.39, *p*_*FDR*_ = 4.37E−03) (Additional file 1: Table S10). Of note, although the DHR residing in the transcription start site (TSS) of *CHRNB1*, of which all probes show hyperhydroxymethylation in the AD cases, did not correlate with *CHRNB1* mRNA expression (*ρ* = − 0.09, *p*_*FDR*_ > 0.05), a linear regression analysis of regressed MTG expression data of *CHRNB1* showed a significant elevation of *CHRNB1* mRNA levels in AD cases (estimate = 0.13, *p* = 1.37E−04) (Additional file 2: Figure S4). For a full transcriptomic investigation of the MTG cohort used in the present study, see the recent publication of Piras et al. [19].

**Table 3:** Differentially methylated, hydroxymethylated, and unmodified regions in the middle temporal gyrus

Gene	Position	Gene feature	*n*	*p* value	Šidák *P*	Average Δ% (range Δ%)
5mC						
*OXT*	chr20:3051954-3052484	TSS; Intron; 5′UTR; CDS	10 (0 up; 10 down)	1.43E−09	1.07E−06	− 3.76 (− 6.94:− 0.43)
5hmC						
*CHRNB1*	chr17:7348322-7348439	TSS; Exon; 5′UTR	5 (5 up; 0 down)	2.63E−07	4.01E−04	1.46 (0.70:1.96)
UC						
*ACTR3C*; *LRRC61*	chr7:150019955-150020946	TSS; Intron; Exon; 5′UTR	17 (1 up; 16 down)	3.54E−12	1.42E−09	− 0.57 (− 1.34:0.02)
*RHBDF2*	chr17:74475240-74475403	Intron; CDS	5 (0 up; 5 down)	1.99E−09	4.85E−06	− 3.45 (− 4.71:− 1.42)
*TMC8*	chr17:76128522-76128907	Intron; CDS	8 (0 up; 8 down)	3.29E−09	3.39E−06	− 1.26 (− 2.84:− 0.26)
*ASPG*	chr14:104551867-104552210	TSS; Intron; 5′UTR; CDS	5 (0 up; 5 down)	1.00E−08	1.16E−05	− 1.21 (− 2.49:− 0.28)
*PIEZO1*	chr16:88844969-88845205	Intron	3 (0 up; 3 down)	1.87E−07	3.14E−04	− 3.08 (− 3.76:− 2.32)
*VWA7*	chr6:31734106-31734472	Intron; CDS	10 (10 up; 0 down)	2.04E−07	2.21E−04	3.39 (2.24:4.23)
*CLMAT3*; *SPARC*	chr5:151066460-151066731	Exon; TSS; 5′UTR	6 (0 up; 6 down)	5.21E−07	7.62E−04	− 0.29 (− 0.64:0.21)
*KIAA1522*	chr1:33231070-33231314	TSS; Exon; 5′UTR; Intron	6 (0 up; 6 down)	8.48E−07	1.38E−03	− 1.85 (− 2.43:− 1.3)
*C3*	chr19:6713227-6713460	Intron; CDS	3 (1 up; 2 down)	9.21E−07	1.57E−03	− 1.20 (− 2.1:0.46)
*PRSS22*	chr16:2908157-2908246	TSS; Exon; 5′UTR	4 (0 up; 4 down)	1.02E−06	4.52E−03	− 1.56 (− 1.91:− 1.39)
*FRAT1*	chr10:99080756-99081017	Exon	3 (3 up; 0 down)	1.50E−06	2.28E−03	2.34 (1.57:3.03)

Differentially methylated (5mC), hydroxymethylated (5hmC), and unmodified (UC) regions in a comparison of Alzheimer’s disease patients (*n* = 45) and controls (*n* = 35). Displayed for each region is the UCSC gene name, chromosomal position (genome build 37), gene feature (TSS, transcription start site; 5′UTR, 5′ untranslated region; CDS, coding sequence), number of probes in region and number of upregulated and downregulated probes (*n*), *p* value and multiple testing-corrected *p* (Šidák-*P*), and average change in beta value (Alzheimer’s disease - control), including the range of the probe differences

Next, a gene regulatory network (GRN) analysis was performed with the unique genes annotated to the 1000 highest ranked probes. Because of different numbers of associated genes from each dataset, we obtained contextualized networks with varying number of interactions. The number of interactions in the contextualized GRNs representing the differential 5mC, 5hmC, and UC MTG states were 325, 398 and 244, respectively. Differential GRN analysis identified several candidate genes highly influential in the simulated transition from a diseased towards a healthy phenotype. Based on a score indicating for each gene, when changed, the number of other genes in the network that were predicted to show altered expression, *IL6* (score = 55), *SIAH1* (score = 78), and *EGF* (score = 55) were found to be the most influential in the 5mC, 5hmC, and UC networks, respectively (Additional file 1: Table S9).

### Blood

Since 5hmC is not enriched in the blood, only BS conversion was used to measure site-specific 5mC levels, also with the HM 450K array. A blood EWAS investigating the association between DNA methylation and conversion to AD was performed at baseline and at follow-up, leading to the identification of 3 differentially methylated positions at baseline and 266 at follow-up (Additional file 1: Tables S4–S6). No significant inflation was detected (Additional file 2: Figure S2; see the “Materials and methods” section for details).

Genomic annotation enrichment analysis of the top sites in blood showed enrichment of mainly intergenic sites (fold enrichment = 1.32, *p* = 5.80E−04) at baseline and proximal promoters (fold enrichment = 0.79, *p* = 1.60E−04) at follow-up (Additional file 2: Figure S9; Additional file 1: Table S8).

The regional analysis found 15 and 21 DMRs associated with conversion to AD at baseline and follow-up, respectively (Table 4; Additional file 2: Figure S5).

**Table 4:** Differentially methylated regions in blood

Gene	Position	Gene feature	*n*	*p* value	Šidák *P*	Average Δ% (range Δ%)
Baseline						
LDLRAD4	chr18:13611370-13611825	TSS; Exon; 5′UTR; Intron	7 (7 up; 0 down)	3.25E−11	2.88E−08	3.64 (2.54:5.14)
ZNF154	chr19:58220080-58220838	TSS; Intron; 5′UTR; CDS; Exon	11 (11 up; 0 down)	1.16E−09	6.16E−07	3.87 (1.75:5.11)
PRRT1	chr6:32116216-32117402	Intron; 3′UTR; CDS	26 (24 up; 2 down)	2.61E−09	8.85E−07	1.77 (− 3.1:5.37)
SYMPK; RSPH6A	chr19:46318514-46319399	Intron; 3′UTR; CDS; TSS; Exon; 5′UTR	7 (7 up; 0 down)	6.18E−09	2.81E−06	3.2 (2.07:4.87)
TENM3	chr4:183728549-183729462	Intergenic	5 (5 up; 0 down)	1.02E−08	4.48E−06	2.69 (− 0.67:4.25)
GLIPR1L2; CAPS2	chr12:75784617-75785296	TSS; Intron; 5′UTR; CDS; Exon	10 (10 up; 0 down)	2.36E−07	1.40E−04	2.72 (1.85:4.5)
GPR35	chr2:241562085-241562758	Intron	6 (6 up; 0 down)	3.16E−07	1.89E−04	3.83 (3.35:4.37)
ZMAT2	chr5:140079591-140080246	TSS; Intron; 5′UTR; CDS	10 (9 up; 1 down)	3.86E−07	2.37E−04	1.21 (− 1.35:2.81)
ZNF649-AS1; ZNF577	chr19:52390810-52391368	Exon; TSS; Intron; 5′UTR	10 (10 up; 0 down)	7.42E−07	5.35E−04	5.11 (2.93:6.61)
ULK1	chr12:132380696-132380904	Intron	3 (3 up; 0 down)	3.57E−07	6.90E−04	2.1 (0.92:3.5)
SLC44A4	chr6:31846769-31847029	TSS; Exon; 5′UTR	8 (0 up; 8 down)	4.61E−07	7.13E−04	− 1.14 (− 3.86:2.41)
OXT	chr20:3051954-3052484	TSS; Intron; 5′UTR; CDS	10 (9 up; 1 down)	9.41E−07	7.14E−04	3.43 (− 0.45:6.79)
FAM222A	chr12:110156245-110156460	Intron; 5′UTR	4 (4 up; 0 down)	2.85E−06	5.33E−03	3.11 (2.66:3.43)
CYBRD1	chr2:172430723-172430817	Intergenic	3 (0 up; 3 down)	1.83E−06	7.78E−03	− 1.75 (− 2.7:− 0.26)
RUNX2	chr6:45391852-45391974	Intron	3 (3 up; 0 down)	5.11E−06	1.67E−02	3.19 (0.74:4.53)
Follow-up						
GSDMD	chr8:144635309-144635611	TSS; Exon; 5′UTR	5 (0 up; 5 down)	7.08E−18	9.43E−15	− 0.68 (− 1.53:− 0.25)
IRGC	chr19:44203583-44203914	Intergenic	3 (0 up; 3 down)	2.40E−12	2.92E−09	0.87 (− 0.32:1.53)
LINC01149	chr6:31409319-31409758	Exon	12 (0 up; 12 down)	3.03E−10	2.78E−07	− 0.96 (− 2.17:− 0.12)
RUFY1	chr5:178986131-178986907	TSS; Exon; 5′UTR; Intron	9 (9 up; 0 down)	3.77E−09	1.96E−06	2.85 (1.3:4.19)
GLIPR1L2; CAPS2	chr12:75784541-75785296	TSS; Intron; 5′UTR; CDS; Exon	11 (11 up; 0 down)	1.30E−08	6.94E−06	1.34 (0.64:2.81)
RAD51B; LOC100996664	chr14:69095057-69095680	Intron; Exon	5 (5 up; 0 down)	3.57E−08	2.31E−05	6.05 (4.89:7.1)
LOC105372397; MAP4K1	chr19:39086733-39087768	Intron; Exon; CDS	5 (4 up; 1 down)	8.14E−08	3.17E−05	− 0.28 (− 1.05:0.3)
KHDRBS2	chr6:62996022-62996703	Exon; TSS; 5′UTR	11 (11 up; 0 down)	6.92E−08	4.09E−05	1.25 (0.69:1.89)
STAG3L5P-PVRIG2P-PILRB; STAG3L5P; PMS2P1	chr7:99933717-99933798	Exon	3 (3 up; 0 down)	1.01E−08	5.02E−05	0.16 (− 0.01:0.45)
ISOC2	chr19:55972646-55973339	TSS; Intron; Exon; 5′UTR	9 (9 up; 0 down)	8.68E−08	5.04E−05	1.52 (0.78:2.51)
RARRES2	chr7:150037988-150038599	Intron; 5′UTR	3 (0 up; 3 down)	1.63E−07	1.07E−04	0.71 (− 0.64:2.19)
LINC01169	chr15:66947171-66947618	Intron	5 (0 up; 5 down)	1.54E−07	1.39E−04	− 2.24 (− 5.41:− 0.83)
TRAM1L1	chr4:118006405-118007226	TSS; CDS	11 (10 up; 1 down)	9.95E−07	4.88E−04	1.14 (− 0.81:2.96)
LDHC	chr11:18433500-18434016	TSS; Intron; Exon; 5′UTR	7 (7 up; 0 down)	6.92E−07	5.40E−04	2.76 (− 0.3:4.7)
ZNF337-AS1; NANP	chr20:25604462-25605179	Intron; Exon; TSS; 5′UTR; CDS	12 (12 up; 0 down)	1.10E−06	6.18E−04	0.4 (− 0.27:1.36)
IFT74; IFT74-AS1	chr9:26956236-26956770	Intron; 5′UTR; TSS; Exon	3 (2 up; 1 down)	8.65E−07	6.52E−04	0.21 (− 0.2:0.52)
MIR3659	chr1:38599626-38600200	Intergenic	4 (0 up; 4 down)	1.34E−06	9.38E−04	− 0.49 (− 1.35:0.64)
LINC01983	chr3:195578011-195578281	Intron	6 (0 up; 6 down)	6.38E−07	9.51E−04	− 0.07 (− 0.76:0.67)
HEXD	chr17:80393124-80393667	Intron; CDS	5 (5 up; 0 down)	1.89E−06	1.40E−03	− 0.52 (− 2.54:0.39)
GNG7	chr19:2543602-2544101	Intron; 5′UTR	5 (5 up; 0 down)	5.29E−06	4.26E−03	1 (0.25:1.95)
F11-AS1	chr4:187422005-187422120	Exon	5 (5 up; 0 down)	1.66E−06	5.81E−03	1.26 (0.4:1.87)

Differentially methylated regions in the comparisons of Alzheimer’s disease converters and non-converters, prior to conversion at baseline (converters *n* = 54, non-converters *n* = 42), and after conversion at follow-up (converters *n* = 41, non-converters *n* = 42). Displayed for each region is the UCSC gene name, chromosomal position (genome build 37), gene feature (TSS, transcription start site; 5′UTR, 5′ untranslated region; CDS, coding sequence), number of probes in region and number of upregulated and downregulated probes (*n*), *p* value and multiple testing-corrected *p* (Šidák *P*), and average change in beta value (Alzheimer’s disease converter - control), including the range of the probe differences

GRNs representing the blood baseline and follow-up states contained 475 and 277 interactions, respectively. Differential GRN analysis identified *WNT3A* (score = 50) as the most influential gene in the baseline network, and *SHH* (score = 33) in the follow-up network (Additional file 1: Table S9).

### Overlap

Only 1 blood DMR, close to *GLIPR1L2*, showed hypermethylation in relation to AD conversion at both the baseline (+2.72%, *p*_***Šidák***_ = 1.40E−04) and follow-up (+ 1.34%, *p*_***Šidák***_ = 6.94E−06) time points. Extracting the probes located in this blood *GLIPR1L2* DMR from the MTG EWAS for comparison showed, in AD cases, lower UC levels (9/10 probes with negative log2 fold change [logFC]), mixed changes for 5mC (6/10 probes with positive logFC), and lower 5hmC levels for the probes that passed the detection threshold (2/2 probes with negative logFC). Even though the UC observations in the MTG are in line with the blood findings, only for one UC probe (cg07311024) the change was nominally significant (logFC = − 0.01, *p* = 3.88E−02). A targeted linear regression analysis of the regressed MTG expression data of *GLIPR1L2* showed a significant decrease in AD cases (estimate = -0.10, *p* = 3.12E−04) (Additional file 2: Figure S6).

**Figure 2.**
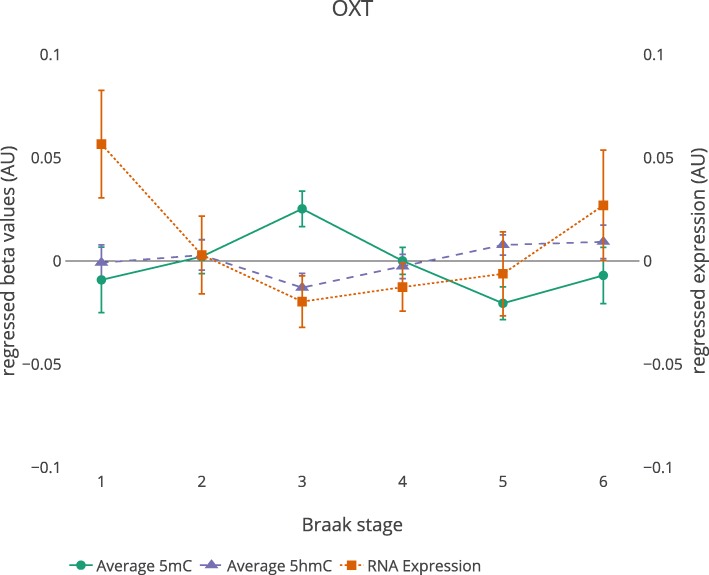
Methylation, hydroxymethylation and expression of *OXT* across Braak staging. Regressed *OXT* expression values and average regressed 5mC and 5hmC values of 10 and 9 overlapping probes within the *OXT* DMR are shown. Regressed values were generated by taking the residuals of a model fitted with the covariates age, gender, and 5 surrogate variables, but excluding the predictor of interest AD diagnosis. Error bars represent mean ± SEM. *N* = 76 for each line.

Interestingly, close to the TSS of *OXT*, we observed a DMR which was detected both in the MTG (− 3.76%, *p*_*Šidák*_ = 1.07E−06), as well as in the blood dataset (at baseline, + 3.43%, *p*_*Šidák*_ = 7.14E−04) (see Additional file 2: Figure S7 for the probe positions of both *OXT* DMRs). MTG *OXT* methylation across Braak stages, as a proxy indicator of disease progression, is displayed in Fig. 2 and suggest *OXT* hypermethylation towards Braak 3-4 stages and *OXT* hypomethylation during later stages. Moreover, in the differential GRN analysis, *OXT* came forward as an influential gene. In case of the 5mC and 5hmC MTG states, a change in *OXT* was predicted to alter the expression of 39 and 54 other genes in the networks, respectively, and in the blood baseline state, *OXT* was predicted to alter 41 genes in the network (Additional file 2: Figures S10–S12; Additional file 1: Table S9).

## Discussion

For the current study, we aimed to identify AD-related changes in epigenetic DNA modifications, comparing brain tissue from AD patients and age-matched controls. In addition, we explored DNA methylation in blood samples from AD-converters and non-converters, both at a preclinical stage and after conversion, identifying an AD-associated DMR in *OXT* in both the brain and blood datasets.

The DHR identified in the MTG resided in the promotor of *CHRNB1*, which encodes acetylcholine receptor subunit beta and is important for cholinergic neurotransmission. In combination with the observed increased levels of *CHRNB1* mRNA in the MTG, this potentially reflects a compensatory mechanism to maintain acetylcholine signaling in AD. Indeed, the acetylcholine-related pathway is known to be altered in AD and, as such, remains an important target in the development of novel treatment options [20]. Previous epigenomic studies of AD using standard BS-conversion have found associations between AD and *RHBDF2* methylation in multiple cortical regions [3, 4]. We replicated these findings; observing an AD-associated DUR in *RHBDF2*, which included the previously detected CpG sites (cg13076843, cg05810363, and cg12163800) and showed the same direction of effect as previously reported. For instance, using conventional bisulfite (BS) conversion, a 3.36% increase in DNA methylation level of cg05810363 has been observed across cortical regions in association with AD neuropathology [3]. Interestingly, a negative correlation between UC levels within the *RHBDF2* DUR and *RHBDF2* mRNA expression was observed in the MTG. *RHBDF2* is thought to be important for the release of tumor necrosis factor, a major inflammatory cytokine associated with neuroinflammation observed in AD [21, 22]. *C3*, another gene with an AD-associated DUR, encodes a central component of the complement system and mediates developmental synapse elimination by phagocytic microglia. *C3* has previously been implicated in mediating synaptic loss in the early stages of AD [23].

The top DMR from the baseline blood analysis, showing hypermethylation in AD, is close to the *LDLRAD4* gene. This gene has previously been associated with schizophrenia and blood pressure and is thought to suppress transforming growth factor (TGF)-β signaling [24–27]. TGF-β is an inflammatory cytokine playing a role in cell survival and synaptic transmission, and various isoforms have been associated with AD [28]. Additional baseline blood DMRs were close to *TENM3*, involved in neurite growth [29], *SYMPK*, involved in polyadenylation regulation of gene expression and which showed increased expression in AD [30], *SLC44A4*, associated with type 1 diabetes mellitus and human aging [31], *ZMAT2*, which had decreased expression in AD [32], *ULK1*, which may play a role in the autophagic degradation of amyloid beta (Aβ) [33], and *RUNX2*, which links bone health and cognitive function and anxiety-like behavior [34]. The DMR that was found both at baseline and follow-up is associated with *GLIPR1L2*. *GLIPR1L2* also showed decreased expression in the MTG. The function of this gene is not well known, but it may play a role in tumor suppression and immune function [35, 36]. The top AD-associated blood DMR at follow-up, showing hypomethylation, is located in *GSDMD*, which encodes a critical factor in pyroptosis; a form a cell death that may be triggered by Aβ [37, 38]. Other genes with a nearby AD-associated blood DMR at follow-up include *KHDRBS2*, previously identified in a genome-wide association interaction analysis in relation to AD [39], *RARRES2*, encoding an adipokine that has been linked to inflammation, obesity, diabetes, and cardiovascular diseases [40], and *GNG7*, for which Braak stage-associated differential methylation has been reported in cortical glial cells of AD patients [41].

Taken together, the observation of epigenetic modifications in several inflammation-associated genes in both brain and blood aligns with the amyloid cascade-inflammatory hypothesis of AD [42]. These findings could reflect either downstream effects resulting from the inflammatory activation seen in AD, or, particularly in the brain, reflect mediating effects of DNA modifications on inflammation as a causative factor. Exploring the exact nature of the AD-associated epigenetic modifications in inflammation-associated genes and the potential for blood biomarkers is thus a pivotal aim for future studies.

Strikingly, our methylomic profiling in MTG and whole blood both led to the identification of a common DMR associated with AD, close to the transcription start site of *OXT*. Our design allowed for the disentanglement of specific 5mC and 5hmC signals in the MTG, which, in the case of *OXT*, suggests they change in opposite directions in relation to AD. The detection of a DMR near *OXT* is in line with a recent report of a nearly identical AD-associated *OXT* DMR (containing 1 additional probe) in the superior temporal gyrus (STG) [43]. This area is located directly above the MTG. Furthermore, using GRN analysis addressing the overlap between the top influential genes in the networks and genes with significant differentially modified regions, we observed *OXT* to consistently appear as one of the most influential genes in both brain and blood GRNs. *OXT* encodes oxytocin, a neuropeptide involved in the neuromodulation of social behavior, stress regulation, and associative learning [44]. Interestingly, the functional impact of *OXT* promoter methylation at the same genomic locus has been recently shown [45]. It was linked to several measures of sociability, superior temporal sulcus activity during social cognition tasks, as well as fusiform gyrus gray matter volume, a brain region closely related to the MTG.

The paraventricular nucleus and supraoptic nucleus are thought to be the main sites of central oxytocin production [46], areas which reportedly undergo cell loss during AD [47]. The remaining neurons are thought to undergo a, potentially compensatory, hypertrophy. One might hypothesize that this activation could initially lead to higher than normal oxytocin levels, before synthesis collapses during the final stages of AD. Interestingly, enhanced levels of hippocampal oxytocin have been associated with memory impairment, and AD-associated elevations of oxytocin have been reported in the hippocampus and temporal cortex [48]. There is also limited evidence oxytocin is reduced in cerebrospinal fluid of manifest AD [49]. Additionally, it has been suggested that co-damage to the locus coeruleus and hypothalamic nuclei could happen early during AD pathogenesis [50], substantiating the hypothesis that oxytocin could serve as an early diagnostic biomarker for AD.

In line with an AD-related increase in temporal cortex oxytocin levels [48], all ten CpG sites within the MTG *OXT* DMR showed decreased levels of methylation in AD cases. Conversely, we observed *OXT* hypermethylation in the DNA from peripheral blood of participants who progressed to dementia. The *OXT* blood DMR was not observed after conversion. Research has shown that independent mechanisms may be involved in peripheral and central regulation of *OXT* expression, supporting this apparent discrepancy observed in blood and brain [51]. Alternatively, these observations suggest there may be a temporal change in *OXT* methylation during AD progression. Looking at MTG *OXT* methylation across Braak stages appears to support the observation of *OXT* hypermethylation at earlier stages, as also seen in the blood, and *OXT* hypomethylation at more advanced stages. Notably, it has recently been reported that oxytocin administration was able to improve social cognition and behavior in frontotemporal dementia patients [52], illustrating the complex modulatory function of oxytocin in different brain regions and its potential use in the treatment of certain manifestations of dementia. Whether oxytocin represents a suitable therapeutic agent for AD remains to be elucidated.

Even though we detect several targets relevant in light of AD, a general lack of overlap between the different analyses presented here might be noted, an observation which is true for EWAS and epigenetics studies in AD in general. Others have discussed a myriad of possible reasons for discrepancies between studies, such as methodological differences, differences in tissue type and processing, study designs, and samples sizes [53]. In view of this, the detection of a common *OXT* DMR in two completely independent cohorts and two different types of tissue, further supported by a recent similar EWAS on the STG [43], makes it an even more promising target for future studies. However, the differences in direction of change and the *OXT* methylation pattern observed over Braak stages indicates these epigenetic changes should be further studied in a longitudinal fashion to establish a clear relationship with AD neuropathology, as well as clinical manifestations of AD.

Given the detection of several regions of interest, it should be noted that the lack of positions significantly associated with AD in the MTG after FDR correction may be the result of a limited sample size. Genome-wide site-specific AD-related epigenetic changes should thus be further investigated using studies with larger sample sizes or meta-analyses. Alternatively, future studies may focus on candidate genes identified in the present work, such as *OXT*.

## Conclusions

Our novel approach confirms some previous epigenetic findings identified in the central nervous system, including *RHBDF2*, as well as revealed novel targets, such as in *CHRNB1*, involving dysregulated DNA hydroxymethylation. Furthermore, the nearly identical *OXT* DMRs found in both the blood and brain suggest a systemic epigenetic dysregulation in AD involving *OXT.* The detection of the *OXT* DMR at pre-dementia stages suggests its potential relevance as a novel biomarker and may offer new treatment strategies to be explored in future studies.

## Materials and methods

### Patients

Informed consent was obtained from all human participants. This includes donors of the Banner Sun Health Research Institute (BSHRI) Brain and Body Donation Program (BBDP), who signed an Institutional Review Board-approved informed consent form, including specific consent to the use of donated tissue for future research [16, 54]. The German Study on Ageing, Cognition and Dementia in Primary Care Patients (AgeCoDe) study protocol was approved by the local ethics committees at the University of Bonn (Bonn, Germany), the University of Hamburg (Hamburg, Germany), the University of Duesseldorf (Duesseldorf, Germany), the University of Heidelberg/Mannheim (Mannheim, Germany), the University of Leipzig (Leipzig, Germany), and the Technical University of Munich (Munich, Germany).

DNA from the MTG was obtained from 82 AD patients and neurologically normal control BBDP donors stored at the Brain and Tissue Bank of the BSHRI (Sun City, AZ, USA) [16, 54] (Table 1). The organization of the BBDP allows for fast tissue recovery after death, resulting in an average post-mortem interval of only 2.8 h for the included samples. Braak staging was carried out for AD neurofibrillary pathology. A consensus diagnosis of AD or non-demented control was reached by following National Institutes of Health AD Center criteria [54]. Comorbidity with any other type of dementia, cerebrovascular disorders, mild cognitive impairment (MCI), and presence of non-microscopic infarcts was applied as exclusion criteria. Although this may limit the generalizability of the current study, these strict exclusion criteria were applied to enhance the detection of AD-specific dysregulation, not confounded by common comorbidities. Detailed information about the BBDP has been reported elsewhere [16, 54].

AgeCoDe is a prospective longitudinal study including 3327 non-demented individuals at baseline, initiated to investigate the early detection of MCI and dementia in primary care [55]. Participants were randomly selected from the general practice registry in six German cities and cognition was assessed at approximately 18-month intervals and 10-month intervals after visit 7, for up to 11 years after baseline. For this study, whole blood DNA was obtained from a subsample of 99 individuals aged above 75 years from this AgeCoDe cohort (Table 2). Of these, 42 were converters: they had no dementia at baseline, had DNA samples available at baseline and follow-up (after ~ 4.5 years), and had sufficient information available for a diagnosis of AD dementia to be made at the 4.5-year follow-up. There were 44 control subjects, who had to adhere to the same criteria, except that they should have no signs of dementia at neither baseline, nor the 4.5-year follow-up, and all subsequent cognitive assessments up to 11 years after baseline. The remaining 13 participants had not yet converted at the 4.5-year follow-up (when blood was drawn), but were diagnosed during a later follow-up, up to a maximum of 11 years after baseline [56]. These samples were grouped together with the other converters.

The groups were matched for age, gender, and *APOE* genotype. The presence of dementia was assessed in all subjects with the Structured Interview for Diagnosis of Dementia of Alzheimer Type, Multi-infarct Dementia, and Dementia of Other Etiology [57] based on the DSM-IV criteria. The dementia diagnosis in subjects who were not personally interviewed was based on the Global Deterioration Scale [58] (≥ 4) and the Blessed Dementia Rating subscales. The etiological diagnosis of AD was based on the criteria of the National Institute of Neurological and Communicative Disorders and Stroke and the Alzheimer’s Disease and Related Disorders Association [59] for probable AD and was only assigned in case of sufficient information provided. All final diagnoses were a consensus between the interviewer and an experienced geriatrician or geriatric psychiatrist. More detailed information about the AgeCoDe cohort has been published previously [55, 56].

### (Hydroxy)Methylomic profiling

For the BBDP samples, the TrueMethyl^TM^ 24 Kit version 2.0 by CEGX^TM^ (Cambridge Epigenetix Limited, Cambridge, UK) was used for BS and oxBS conversion of genomic DNA (gDNA) extracted from frozen MTG tissue. All laboratory procedures were performed at GenomeScan (GenomeScan B.V., Leiden, the Netherlands), without knowledge of the phenotypic characteristics of the samples and according to the manufacturer’s instructions. Prior to conversion, high molecular weight (HMW) gDNA was quantified using a PicoGreen assay (Invitrogen, Carlsbad, CA, USA), and gel-electrophoresis was performed to assess gDNA quality. All samples were of sufficient quantity and quality. A volume of 1 μg HMW gDNA was used per sample, which, after purification and denaturation, was split into two samples that underwent either DNA oxidation (oxBS samples) or mock DNA oxidation (BS samples). Subsequently, all samples were BS-treated, and the yield of the samples was assessed by a Qubit ssDNA assay (Invitrogen). An additional quality control, using a restriction enzyme only able to cut unconverted cytosines, was performed for a qualitative assessment of 5hmC oxidation and BS conversion. From each BS/oxBS-treated DNA sample, 8 μL was amplified and hybridized on HM 450K arrays (Illumina, Inc., San Diego, CA, USA), and the Illumina iScan was used for imaging of the array. Sample preparation, hybridization, and washing steps for the Illumina Infinium Methylation Assay of the BeadChip arrays were performed according to the manufacturer’s protocol.

For the AgeCoDe samples, gDNA was isolated from whole blood and DNA concentration and purity was determined using the NanoDrop ND1000 spectrophotometer (Thermo Fisher Scientific). All samples were of sufficient quantity and quality. Five hundred nanograms of gDNA was used for BS conversion, using a Qiagen EpiTect 96 Bisulfite Kit (Qiagen, Hilden, Germany) according to the manufacturer’s protocol. A total of 200 ng of BS converted DNA was analyzed using HM 450K arrays according to the manufacturer’s instructions. The Illumina iScan was used for imaging of the array.

### Transcriptomic profiling

Total RNA extracted from frozen MTG, from matched samples as used for the epigenetic MTG analyses, was isolated with the RNeasy Mini Kit (Qiagen) starting with at least 60 mg of tissue. Raw expression data was obtained at the BSHRI, using the HumanHT-12 v4 BeadChip (Illumina).

### Statistical analysis

All computational and statistical analyses were performed using the statistical programming language R (version 3.3.2) [60] and RStudio (version 1.0.136) [61], unless otherwise specified. Raw IDAT files from the Illumina iScan were loaded into R using the *minfi* package (version 1.20.2) [62]. To confirm that the longitudinal samples were from the same donor a genetic fingerprinting test was performed based on the 65 SNP probes included on the HM 450K chip, as implemented in the *ewastools* package [63]. Based on this test, 2 donors with mismatching samples were detected and excluded from the blood data. Next, the gender of the samples was predicted based on X chromosome methylation using the *DNAmArray* package (version 0.0.2) [64], compared with the assumed gender, and mismatches were excluded (1 mismatched sample was excluded from the blood data). Cross-hybridizing probes and probes containing a common SNP in the sequence or within 10 bp of the sequence were removed [65]. The “pfilter” function of the *wateRmelon* package (version 1.18.0) [66] was used for probe filtering (6 969 and 1 437 probes were removed from the MTG and blood data, respectively). The remaining probe data was normalized using the *dasen* method, as implemented in the *wateRmelon* package [66]. Probes on the X and Y chromosomes were excluded from further analyses.

Following normalization, two sets of beta values, from the standard BS arrays (5mC + 5hmC) and from the oxBS arrays (5mC), were generated in case of the MTG. By subtracting oxBS beta values from the BS beta values (Δβ_BS-oxBS_) for each probe in each sample, 5hmC levels were calculated (Fig. 1). UC values were determined as 1-BS (1-β_BS_). It should be noted that other DNA demethylation intermediates, such as 5-formylcytosine (5fC) and 5-carboxylcytosine may be represented in the BS or UC levels, as it is currently unclear how these intermediates respond to oxBS conversion [67]. However, these intermediates are present at very low levels and are not enriched in brain tissue like 5hmC is [68]. In order to reduce noise and filter out non-hydroxymethylated sites, outliers deviating more than ± 2SD from the probe mean in the 5hmC dataset were determined and set to the mean ± 2SD first, and subsequently, a threshold of zero was applied to the mean of individual probes (218,009 5hmC values were excluded). Boxplots and density plots of raw and normalized beta values per sample were inspected for clear outliers (2 MTG samples were excluded due to clear deviation from the other samples; data not shown). After data processing, 80 MTG and 96 blood samples remained, with 396,600 remaining probes for MTG 5mC and UC, 178,591 5hmC MTG probes, and 402,480 remaining probes in the blood datasets. The case-control analysis of the blood baseline data included all 96 samples (54 converters, 42 controls), while follow-up data included 83 samples, including the 41 converters that had already converted to AD at the 4.5-year follow-up and excluding those that had converted later. All individuals in the follow-up analysis were also included in the baseline analysis.

An initial model with beta values as outcome, AD diagnosis/conversion as predictor, and age and gender as covariates was used for a surrogate variable (SV) analysis with the *sva* package (version 3.22.0) [69]. The first 5 SVs of this analysis were added to the model to adjust for unobserved confounders, including potential batch effects and differences in cell type composition. As the addition of SVs still resulted in inflation of the regression statistics (lambda = 1.43) of the blood follow-up analysis, and none of the SVs strongly correlated with the HM 450K chip IDs (which was the case for the other analyses), the chip IDs were also added to the model for this analysis. This successfully eliminated the inflation (lambda = 1.00).

Linear regression was performed using the *limma* package (version 3.30.11) [70] to test the association between the beta values and AD diagnosis/conversion. Test statistics were adjusted for bias and inflation with the *bacon* package (version 1.2.0) [71]. An FDR correction for multiple testing was applied to the *p* values to identify differentially (hydroxy)methylated and unmodified positions (probes with *p*_*FDR*_ < 0.05). Individual probes were annotated using Illumina UCSC annotation.

To examine the distribution of 5mC, 5hmC, and UC levels across genomic regions, we annotated the 1000 highest ranking probes (Additional file 1: Tables S2–S7) using ENCODE annotation data, as described by Slieker et al. [72]. Fisher’s exact test was used to assess enrichment in specific genomic regions.

To identify differentially (hydroxy)methylated and unmodified regions (DHRs/DMRs/DURs), spatial correlations between *p* values of the association analysis were determined using *comb-p* [73] with a seeding *p* value of 0.01 and a window size of 1000 bp. Obtained *p* values were Stouffer-Liptak-Kechris corrected for adjacent *p* values and were subsequently corrected for multiple testing using the Šidák correction. Of the regions detected by *comb-p*, only those containing at least 3 CpGs and having a *p*_*Šidák*_ < 0.05 were accepted as differentially modified regions.

GRNs have been extensively used to achieve deeper understanding of disease related mechanisms [74]. Different topological characteristics of these networks, such as connectivity of nodes [75] or gene-gene interaction tendency in cell/tissue specific contexts [76], have been used to predict disease-related genes. Here, we have employed an in-house developed differential GRNs inference approach [77], which relies on gene expression data to infer GRNs specific to a given gene expression program. The initial set of interactions among the genes of interest was compiled from literature-based database ARIADNE [78] and consists of interactions belonging to the categories of “Direct Regulation,” “Expression,” and “Promoter Binding.” The obtained set of interactions is not context-specific as they are reported to happen in different cell/tissue types and organisms. To obtain context-specific networks from the literature interaction maps, the pruning of interactions incompatible with the gene expression state was carried out, which resulted in contextualized networks compatible with the given gene expression state of the system. As a differential expression setting was used here, we obtained two contextualized GRNs for each state, representing the different network topology of diseased and healthy phenotype. The differential network topology helps us in identifying the set of genes that are regulated by different transcription factors in both networks. These genes formulate an ideal set of candidate perturbagens, as to change their expression state we have to perturb them individually. The obtained contextualized networks were used to identify genes in the common elementary circuits (positive and negative circuits) that can also serve as a set of candidate genes for perturbation. Genes in elementary circuits have been reported to play a crucial role in maintaining network stability [79] and are considered as a necessary condition for a network to have an attractive cycle [80]. In this regard, genes present in the common elementary circuits are considered to be the backbone of the network and any perturbations in the expression levels of these genes might lead the system to deviate from the normal steady state of the system, which can be described as a transition from healthy to a diseased state. Once we obtained a set of optimal perturbation candidates, we performed single-gene perturbation simulations to see the effect of change in expression of a single gene on all the other genes in the GRN. This measure tells us about the influential capability of the selected gene in the network; the higher the number of downstream genes being affected by perturbing a candidate gene, the more crucial is its role in the regulation of other genes in the GRN.

Positions from the AD association analyses were ranked based on a combined *p* value and log2 fold change ranking score. The GRN analysis was then conducted separately for the genes annotated to the 1000 highest ranked sites in the MTG (5mC, 5hmC, and UC separately) and blood (baseline and follow-up separately) (Additional file 1: Tables S2–S7). Closest UCSC TSS annotation was used to obtain unique genes. After applying the differential GRN analysis on the contextualized networks, we ranked the key candidate genes based on their scores. This score represents the number of genes whose gene expression is changed (shifted from diseased towards the healthy phenotype) upon perturbation of the candidate gene.

Raw RNA expression data was exported from Illumina’s GenomeStudio (version 2011.1) with the Expression Module (v1.9.0) for further analysis in R. Of the 80 subjects used for the epigenetic analyses, 1 case was not included on the expression array, and 3 additional cases were excluded after quality control of the data, due to extreme outlying values or failed reads, leaving 76 subjects for further analyses. Data was quantile-quantile normalized. Using the same model as for the regression analysis, the sva package was used to determine SVs for the epigenetic and expression datasets. The effects of age, gender, and 5 SVs were regressed out of the epigenetic and expression data using *limma* (i.e., “regressed data” refers to the residuals of a model fitted with the covariates, excluding the predictor of interest, being AD diagnosis or conversion in this case). Spearman correlations were determined for the expression data and the average of the regressed beta values of the probes in the DMRs, DHRs, and DURs, as well as correlations between the different epigenetic markers (5mC, 5hmC, and UC) for these probes, using the Hmisc package (version 4.0-2) [81].

## Supplementary information

**Additional file 1.** All Supplementary Tables (1–10), including descriptions.

**Additional file 2.** All Supplementary Figures (1–12) and descriptions.

## Data Availability

The datasets generated from the BSHRI-BBDP samples and analyzed during the current study are available in the Gene Expression Omnibus (GEO; https://www.ncbi.nlm.nih.gov/geo/) repository, under GEO accession numbers GSE109627 and GSE109887 for the epigenetic and expression data, respectively. The datasets generated from the AgeCoDe samples and analyzed during the current study are not publicly available as participants did not provide informed consent for this, but are available from the corresponding author on reasonable request.

## Abbreviations

5fC: 5-Formylcytosine
5hmC: 5-Hydroxymethylcytosine
5mC: 5-Methylcytosine
AD: Alzheimer’s disease
AgeCoDe: Study on Ageing, Cognition and Dementia in Primary Care Patients and Stroke and the Alzheimer’s Disease and Related Disorders Association
Aβ: Amyloid beta
BBDP: Brain and Body Donation Program
BS: Bisulfite
BSHRI: Banner Sun Health Research Institute
DHR: Differentially hydroxymethylated region
DMR: Differentially methylated region
DUR: Differentially unmodified region
EWAS: Epigenome-wide association study
FDR: False discovery rate
gDNA: Genomic DNA
GRN: Gene regulatory network
HM 450K array: Illumina’s Infinium HumanMethylation450K microarray
HMW: High molecular weight
logFC: log2 fold change
MCI: Mild cognitive impairment
MTG: Middle temporal gyrus
oxBS: Oxidative BS
STG: Superior temporal gyrus
SV: Surrogate variable
TGF: Transforming growth factor
TSS: Transcription start site
UC: Unmodified cytosine
