# Supplementary material for: Alzheimer’s disease-associated (hydroxy)methylomic changes in the brain and blood
Source: Clin Epigenetics. 2019 Nov 27;11:164. doi: 10.1186/s13148-019-0755-5 (PMC6880587; doi:10.1186/s13148-019-0755-5)
Supplement: Supplementary file 2 — Additional file 2. All Supplementary Figures (1–12) and descriptions. [file 13148_2019_755_MOESM2_ESM.pdf]

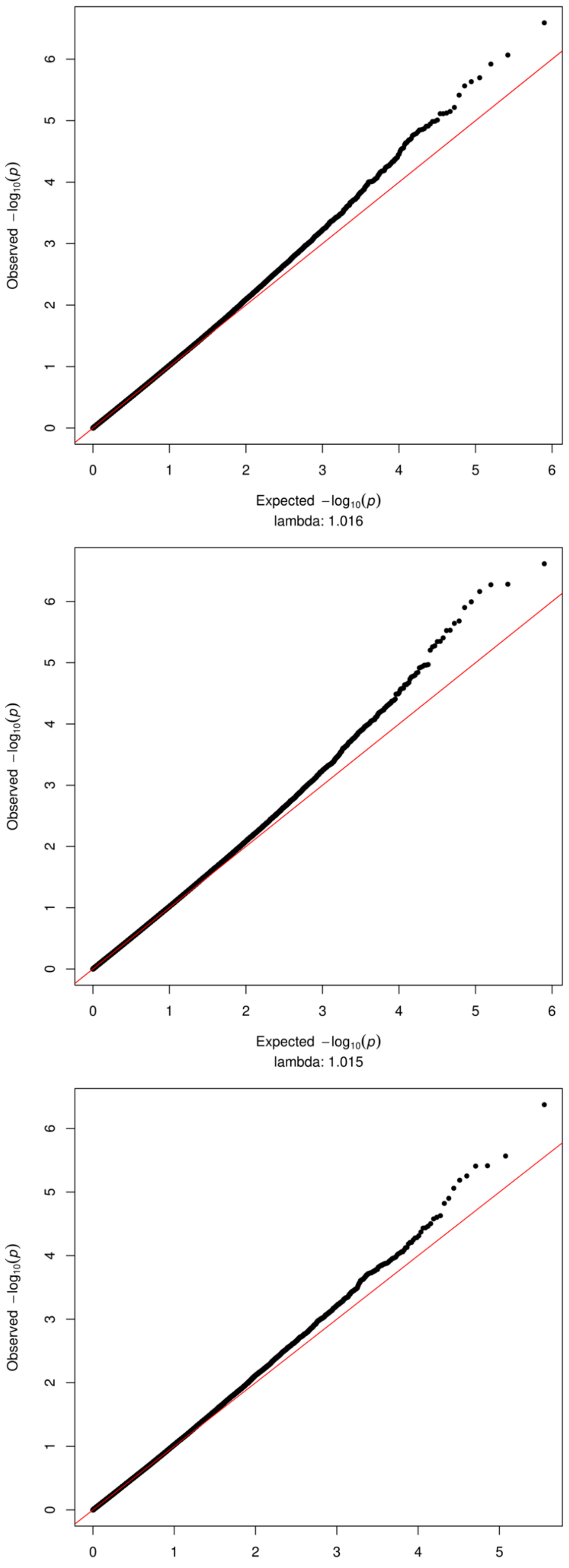

Supplementary Figure 1. QQ plots of the unmodified cytosine (UC; top), 5-methylcytosine (5mC; middle), and 5-hydroxymethylcytosine (5hmC; bottom) regression analyses of middle temporal gyrus tissue.

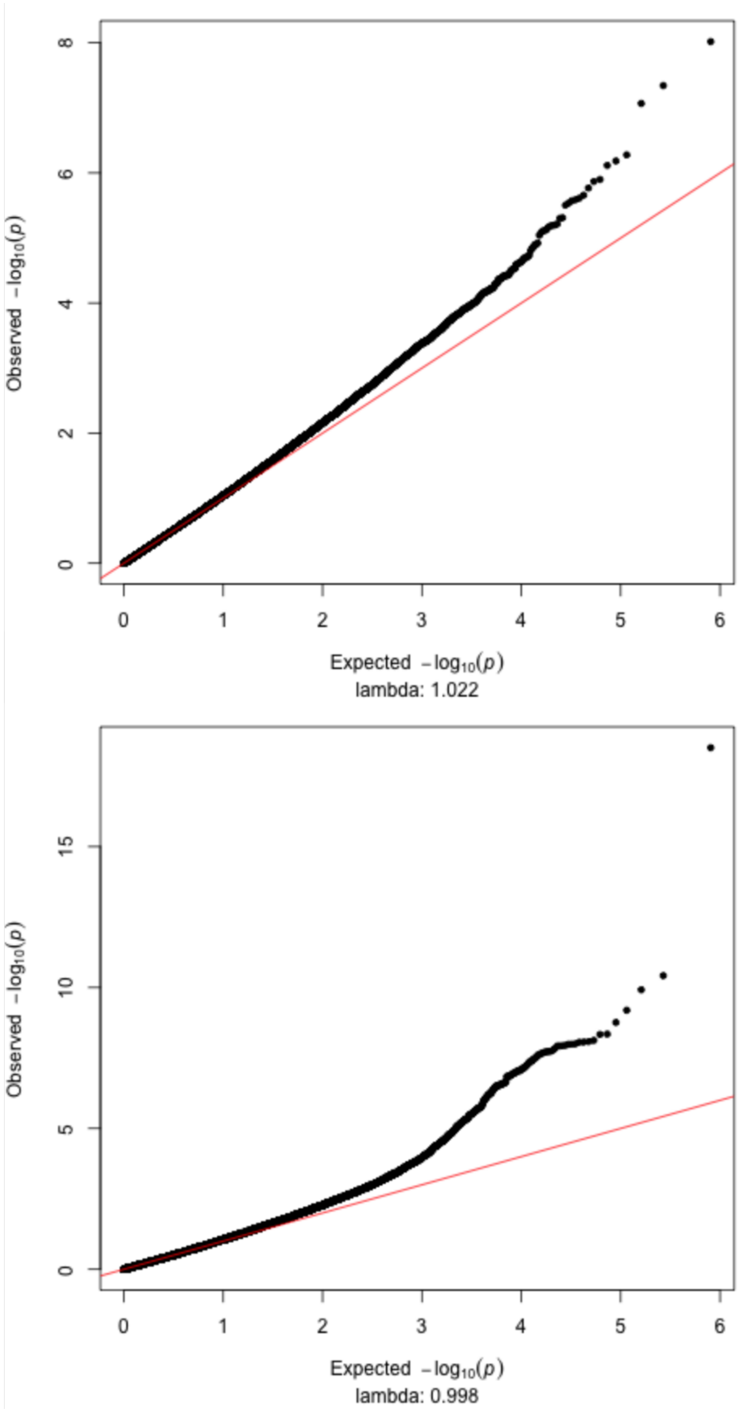

Supplementary Figure 2. QQ plots of the 5-methylcytosine (5mC) baseline (top) and follow-up (bottom) regression analyses of blood.

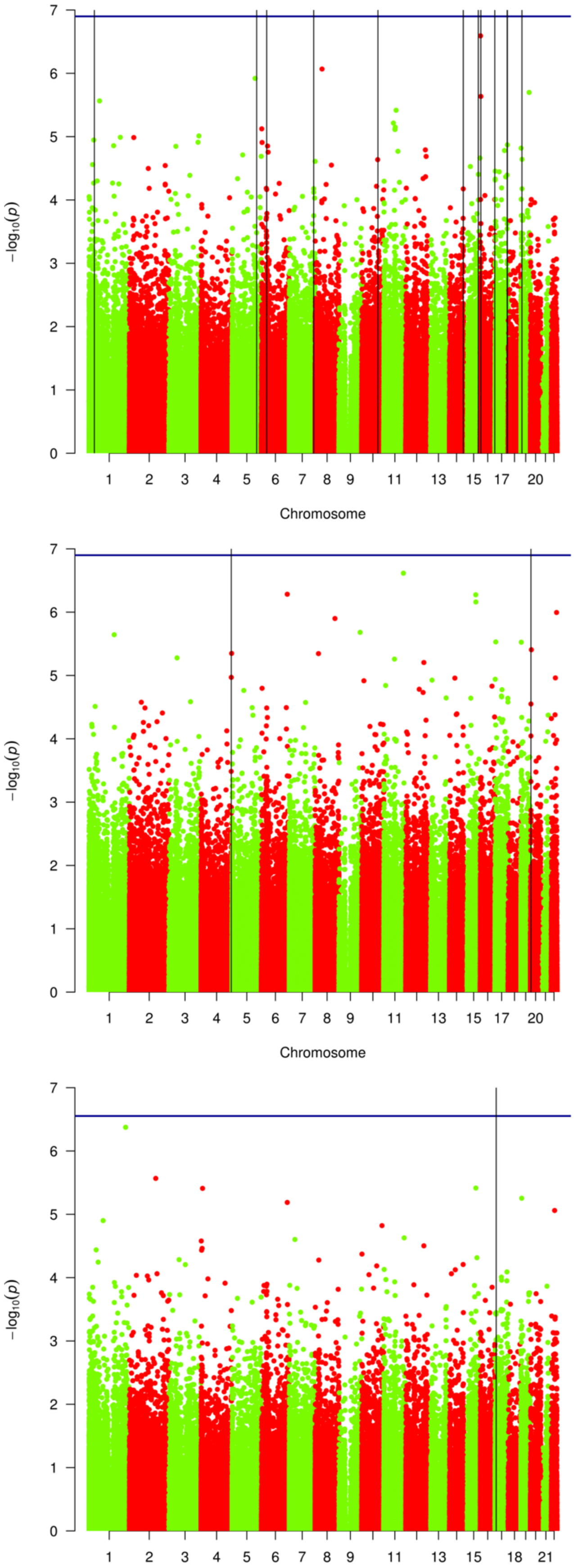

Supplementary Figure 3. Manhattan plots of the unmodified cytosine (UC; top), 5-methylcytosine (5mC; middle), and 5-hydroxymethylcytosine (5hmC; bottom) regression analyses of the middle temporal gyrus. The blue line in the Manhattan plots indicates the genome-wide significance threshold based on the number of probes included in the analysis. Black lines indicate the locations of differentially modified regions (see Table 1 for more details).

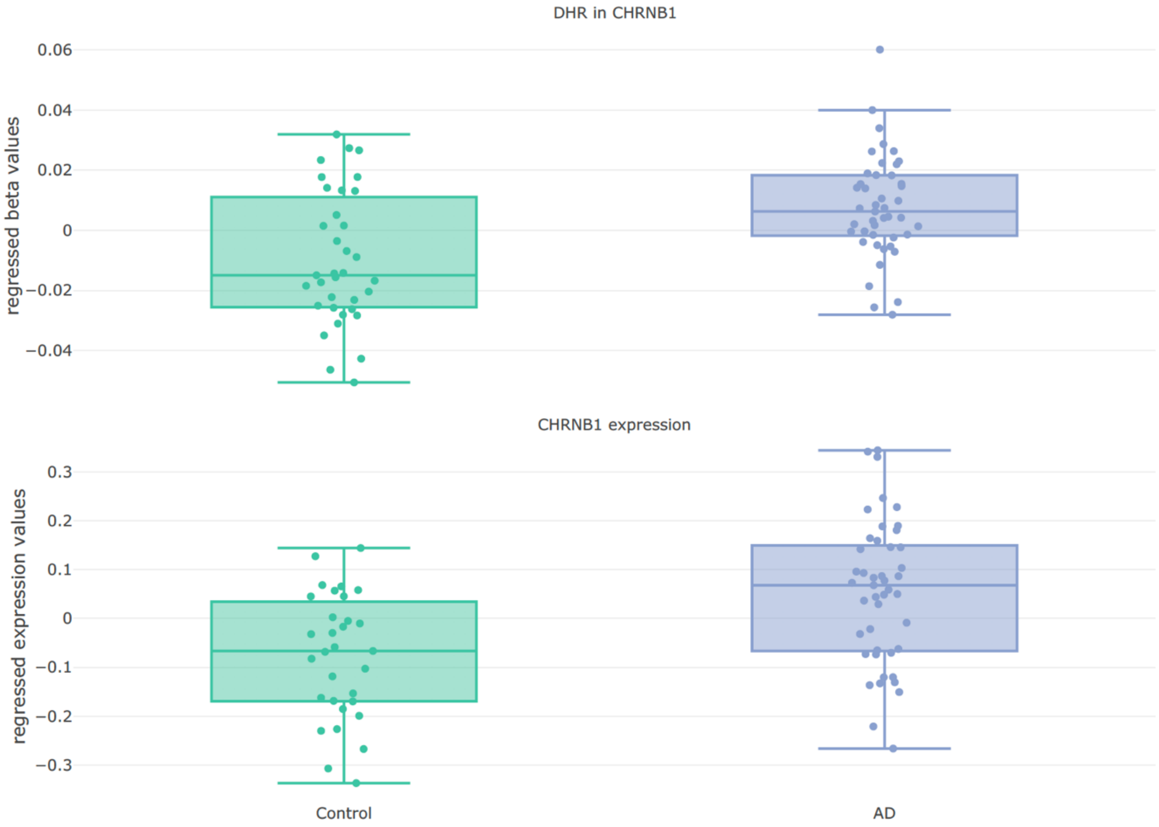

Supplementary Figure 4. Boxplots of the mean regressed 5-hydroxymethylcytosine (5hmC) values of the probes located in the differentially hydroxymethylated region inside *CHRNB1* (top) and regressed expression values of *CHRNB1* (bottom) for Alzheimer's disease (AD) patients and controls. The beta and expression values were regressed using the same covariates as used for the epigenome-wide association analysis. Note that not all 80 samples used for epigenetic profiling were included on the expression array (control n = 31, AD n = 45).

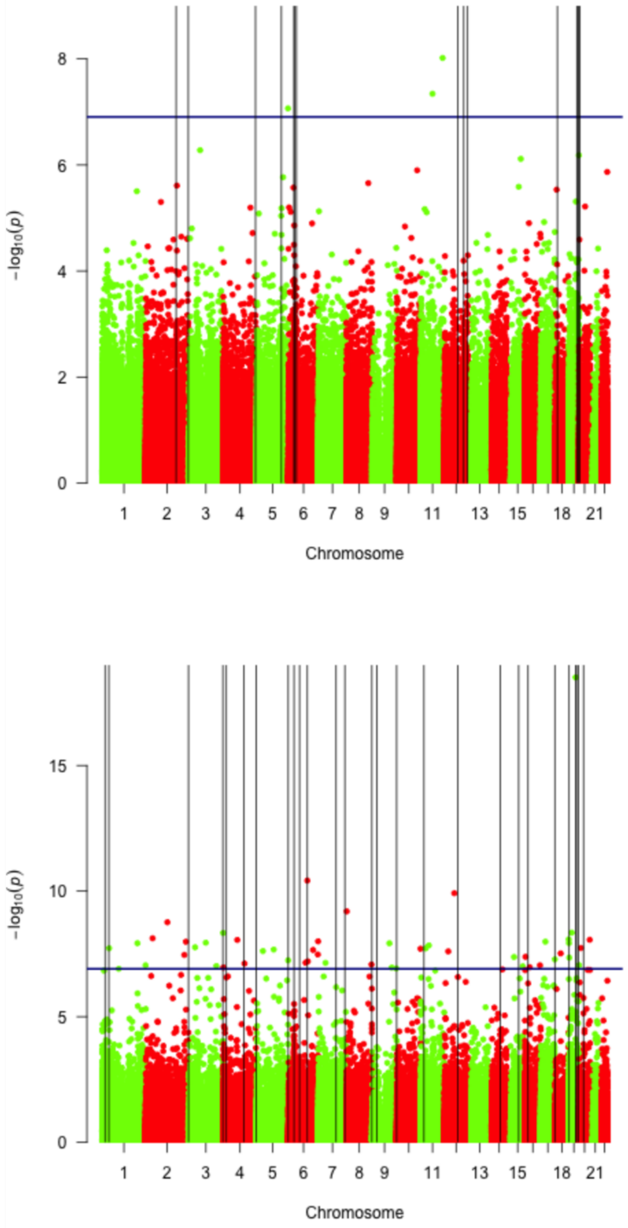

Supplementary Figure 5. Manhattan plots of the 5-methylcytosine (5mC) regression analyses of blood at baseline (top) and follow-up (bottom). The blue line in the Manhattan plots indicates the genome-wide significance threshold based on the number of probes included in the analysis. Black lines indicate the locations of differentially modified regions (see Table 2 for more details).

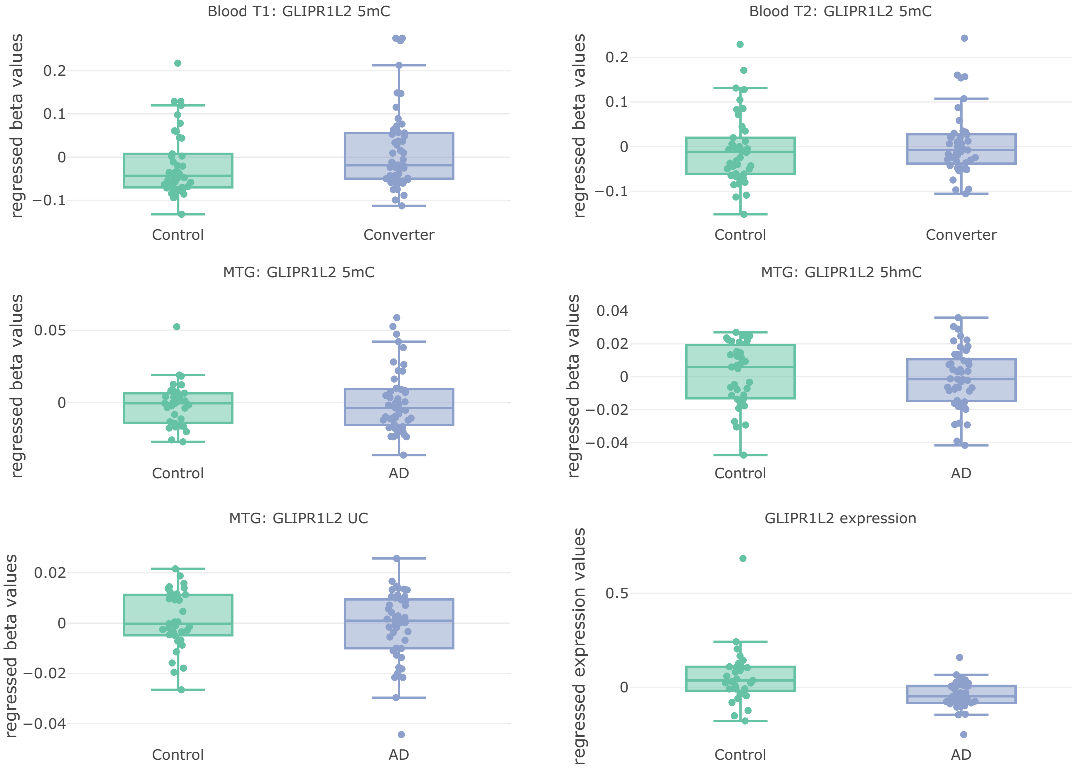

Supplementary Figure 6. Boxplots of the mean regressed 5-methylcytosine (5mC) values of the probes located in the differentially methylated region (DMR) inside *GLIPR1L2* that was detected in the blood (means are based on the 10 probes that were included in the DMR both at baseline [T1] and follow-up [T2]), for converters to AD dementia and controls (top row). Although this DMR was only detected in the blood, 5mC, 5-hydroxymethylcytosine (5hmC) and unmodified cytosine (UC) values are also shown for the middle temporal gyrus (MTG) for Alzheimer's disease (AD) patients and controls (middle row, bottom left). Regressed expression values of *CHRNB1* are shown on the bottom right. The beta and expression values were regressed using the same covariates as used for the epigenome-wide association analysis. Note that not all 80 samples used for epigenetic profiling were included on the expression array (control n = 31, AD n = 45).

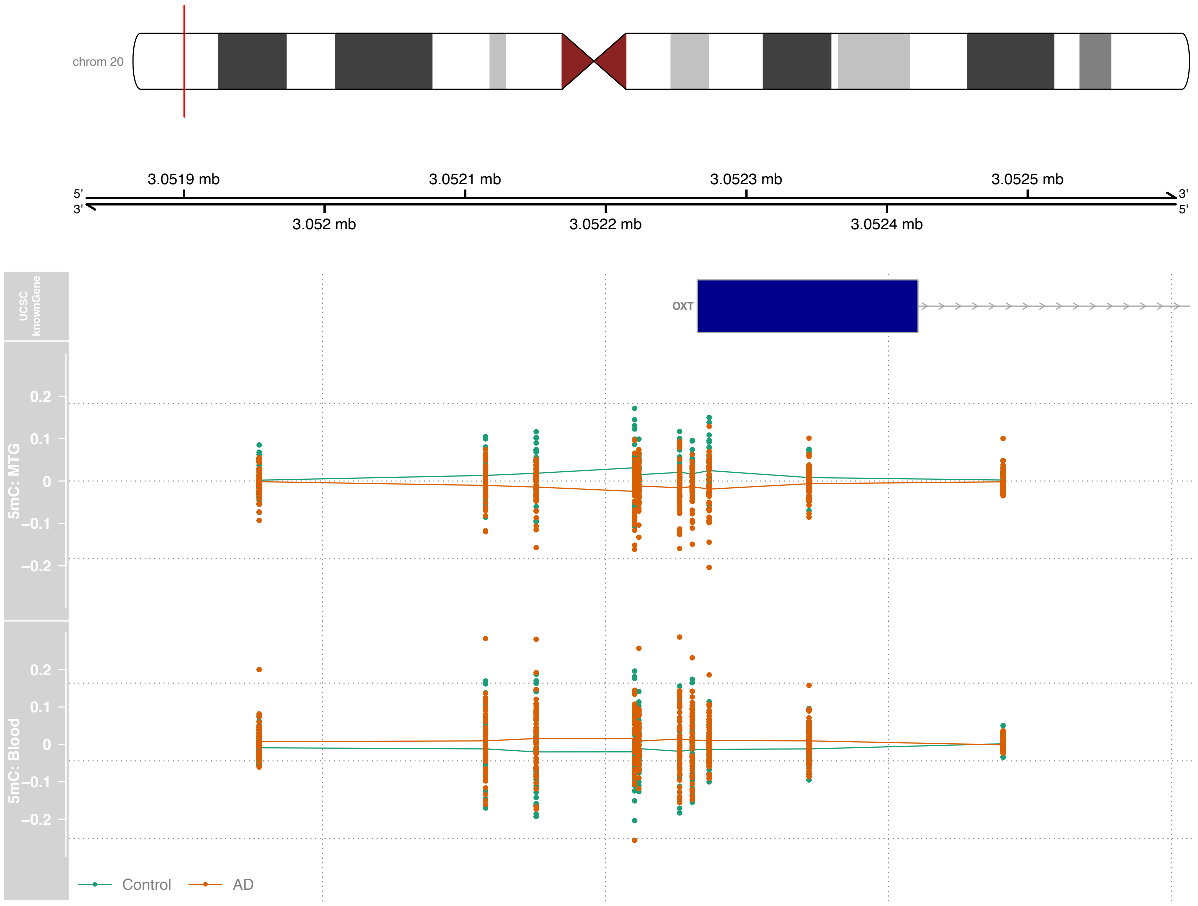

Supplementary Figure 7. Genomic location of the probes constituting the differentially methylated regions (DMRs) in the *OXT* transcription start site (TSS) identified in the middle temporal gyrus (MTG) and blood. The position in chromosome 20 is shown on top and the UCSC knownGene track shows the first exons of *OXT*. The lower two tracks show the position and regressed 5-methylcytosine (5mC) beta values of the probes inside the DMRs, grouped by Alzheimer's disease (AD) patients and controls. Beta values were regressed using the same covariates as used for the epigenome-wide association analysis.

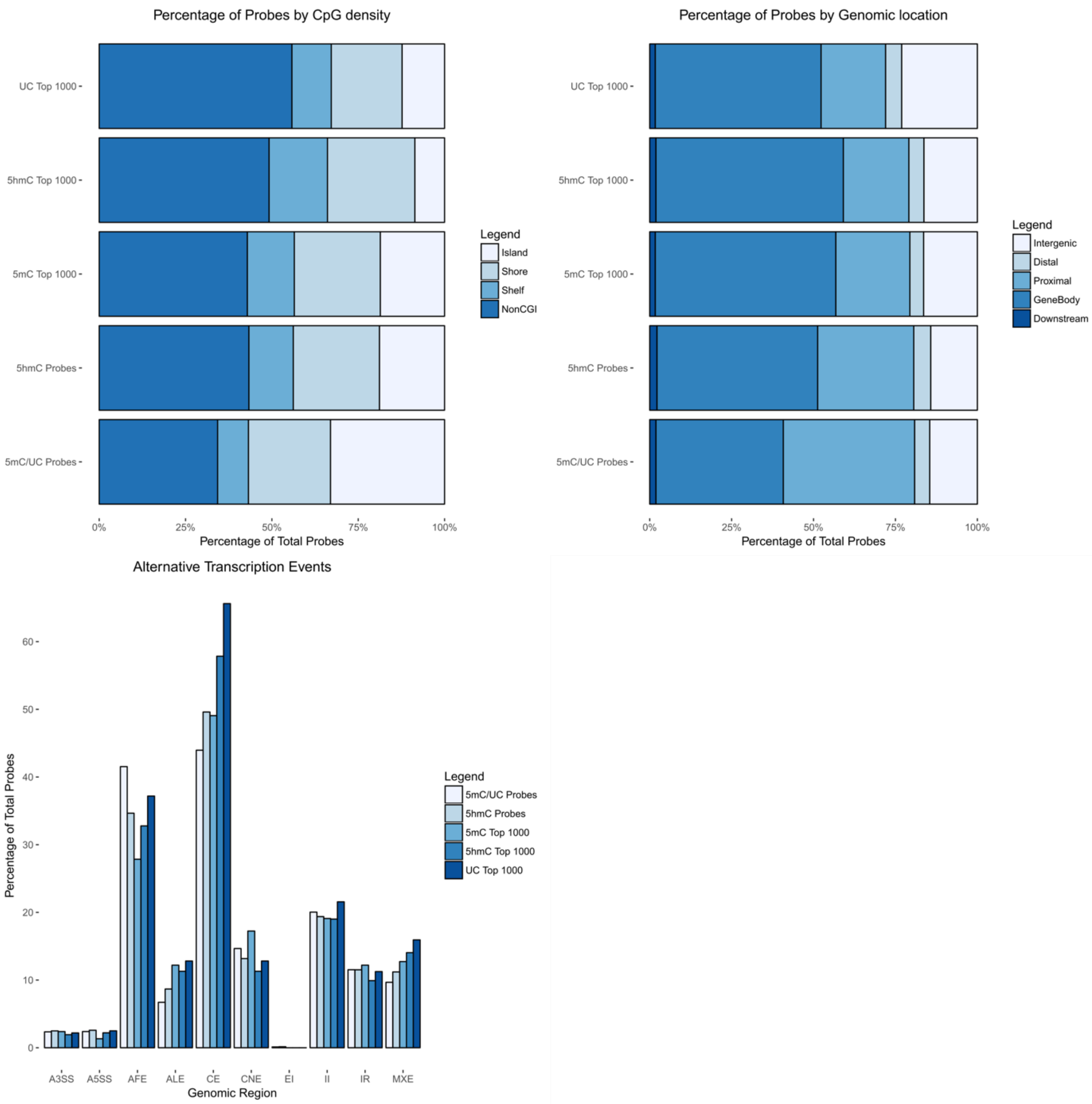

Supplementary Figure 8. Distribution of probes across CpG densities in the middle temporal gyrus (top left). Displayed are the distributions of all 5mC and UC probes, all 5hmC probes passing threshold, and the top 1000 AD-associated 5mC, 5hmC and UC probes across CpG islands, shores, shelves and non-CGI regions. Distribution of probes across genomic locations in the middle temporal gyrus (top right). Displayed are the distributions of all 5mC and UC probes, all 5hmC probes passing threshold, and the top 1000 AD-associated 5mC, 5hmC and UC probes across intergenic regions, distal promoters, proximal promoters, gene bodies, and downstream regions. Distribution of probes across functional genomic regions in the middle temporal gyrus (bottom left). Displayed are the distributions of all 5mC and UC probes, all 5hmC probes passing threshold, and the top 1000 AD-associated 5mC, 5hmC and UC probes. A3SS: Alternative 3’ splice site; A5SS: Alternative 5’ splice site; AFE: Alternative first exon; ALE: Alternative last exon; CE: Cassette exon; CNE: Constitutive exon; EI: Exon isoforms; II: Intron isoforms; IR: Intron retention; MXE: Mutually exclusive exon.

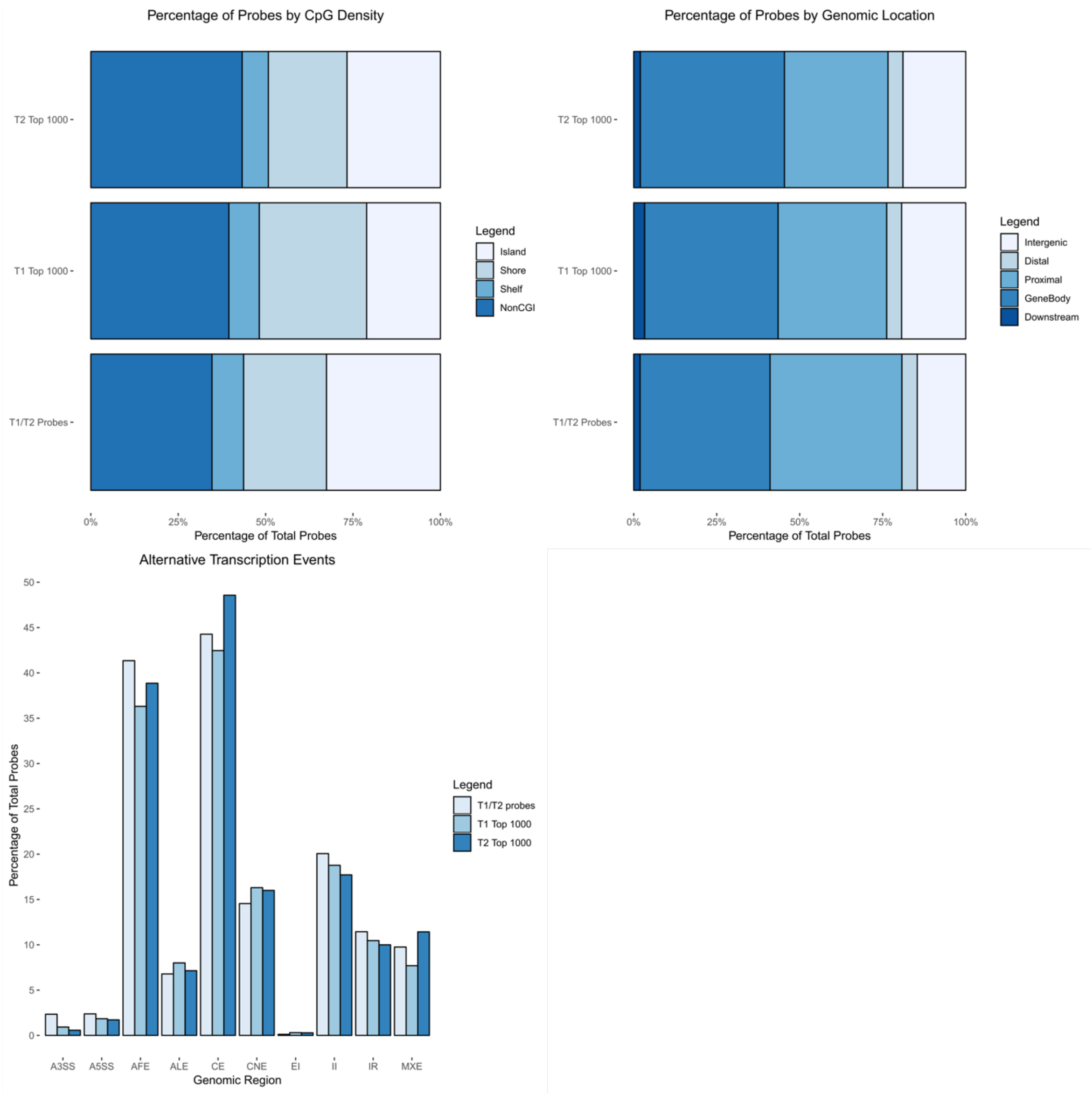

Supplementary Figure 9. (top left) Distribution of probes across CpG densities in blood. Displayed are the distributions of all methylated probes, and the top 1000 AD-associated methylated probes across CpG islands, shores, shelves and non-CGI regions at baseline (T1) and follow-up (T2). (top right) Distribution of probes across genomic locations in blood. Displayed are the distributions of all methylated probes, and the top 1000 AD-associated methylated probes across intergenic regions, distal promoters, proximal promoters, gene bodies, and downstream regions at baseline (T1) and follow-up (T2). (bottom left) Distribution of probes across functional genomic regions in blood. Displayed are the distributions of all 5mC probes, and the top 1000 AD-associated 5mC probes at baseline (T1) and follow-up (T2). A3SS: Alternative 3' splice site; A5SS: Alternative 5' splice site; AFE: Alternative first exon; ALE: Alternative last exon; CE: Cassette exon; CNE: Constitutive exon; EI: Exon isoforms; II: Intron isoforms; IR: Intron retention; MXE: Mutually exclusive exon.

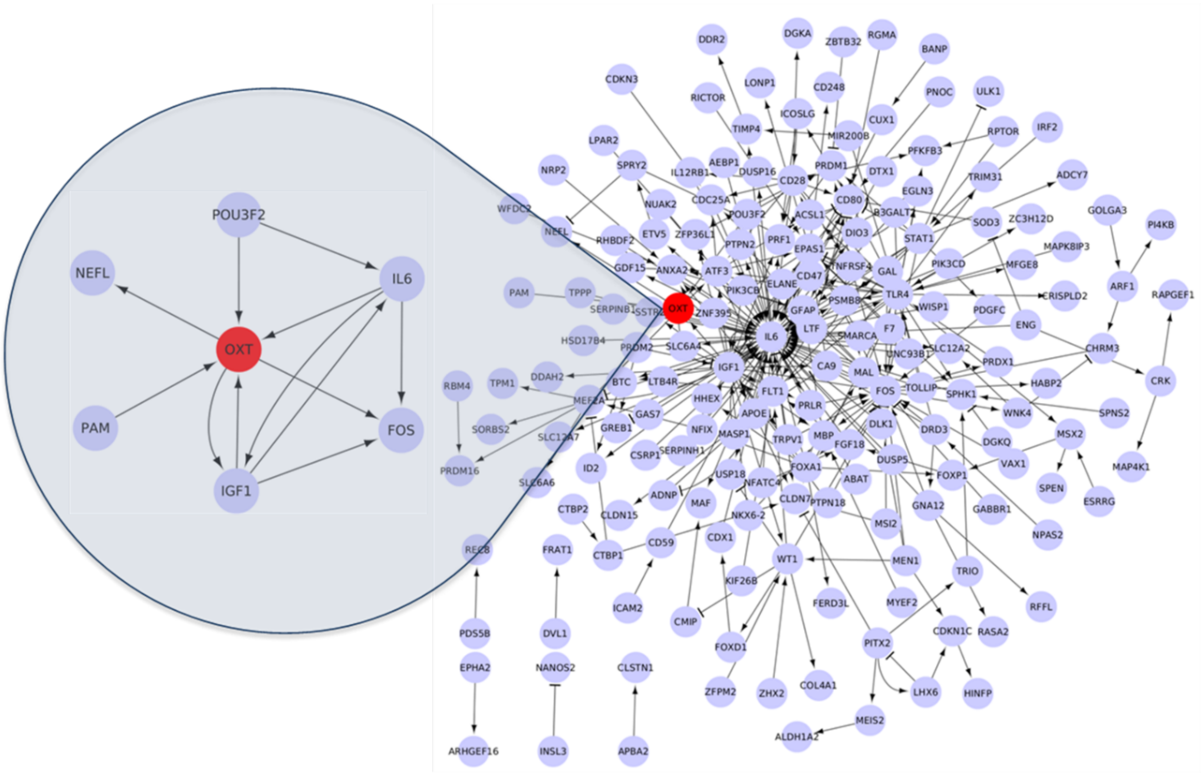

Supplementary Figure 10. *OXT* as a key perturbation candidate in the network representing the 5mC state in the middle temporal gyrus.

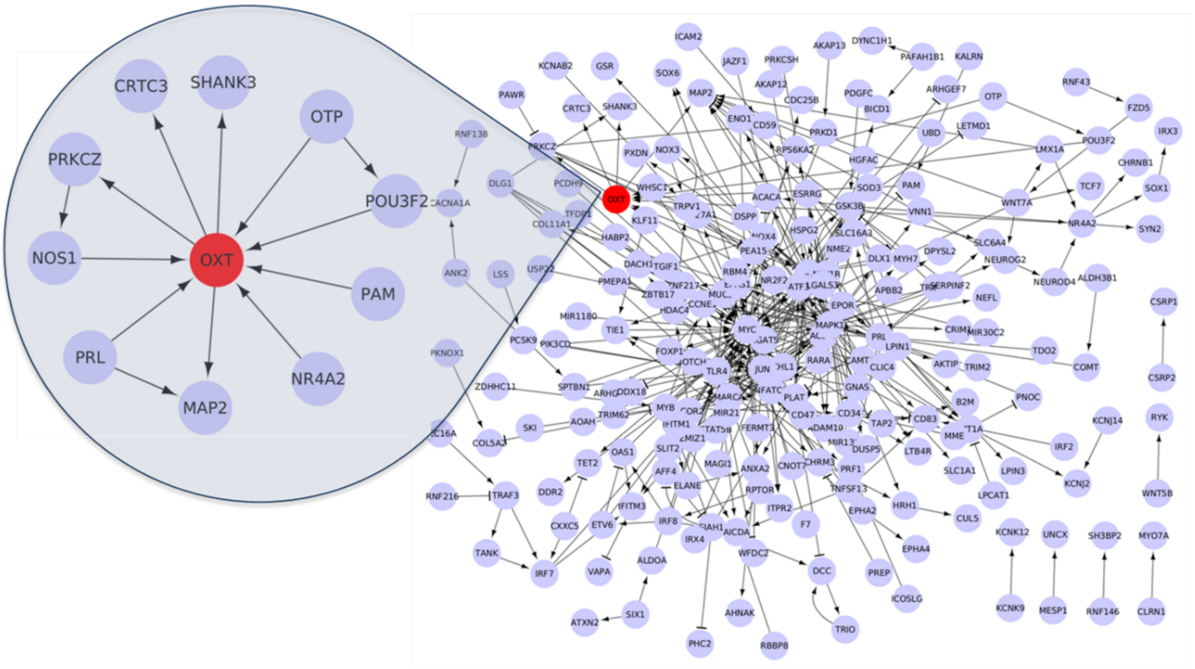

Supplementary Figure 11. *OXT* as a key perturbation candidate in the network representing the 5hmC state in the middle temporal gyrus.

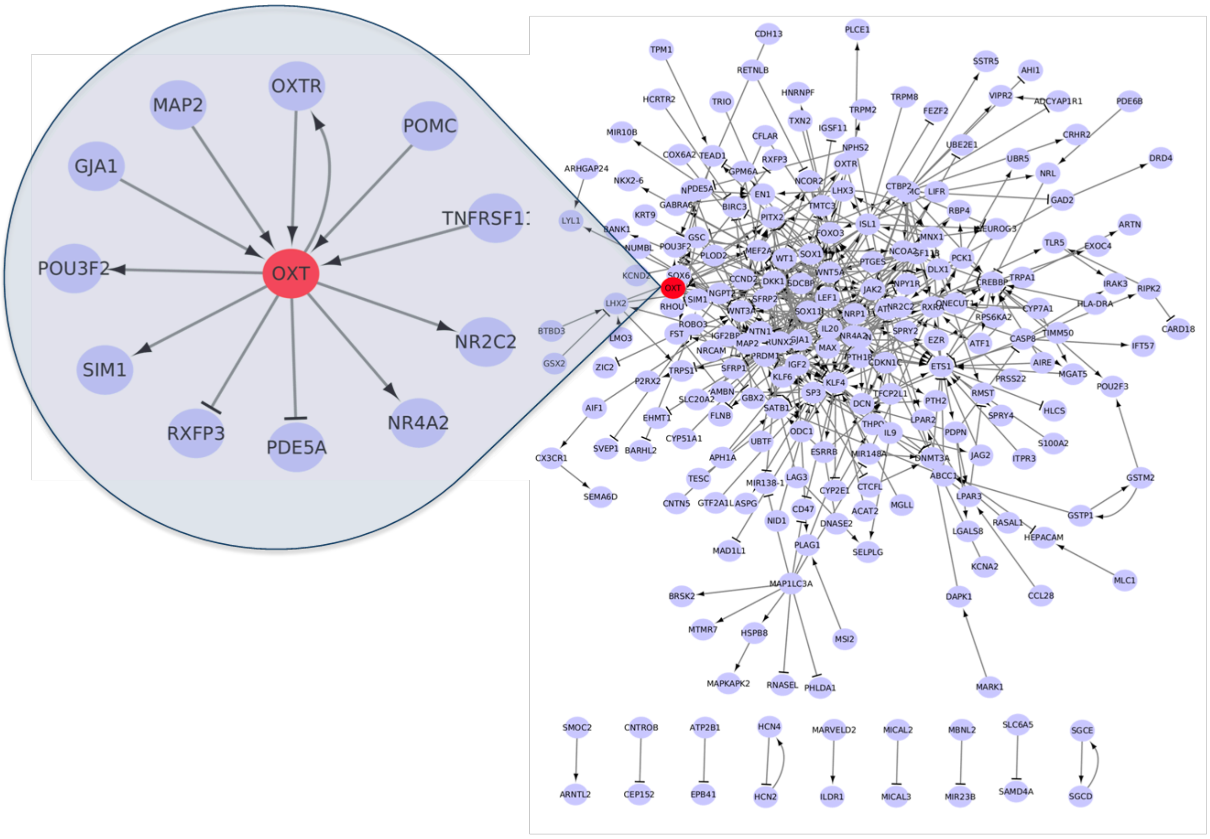

Supplementary Figure 12. *OXT* as a key perturbation candidate in the network representing the 5mC state in blood baseline (T1).
